# Interaction between endothelial nitric oxide synthase rs1799983, cholesteryl ester-transfer protein rs708272 and angiopoietin-like protein 8 rs2278426 gene variants highly elevates the risk of type 2 diabetes mellitus and cardiovascular disease

**DOI:** 10.1186/s12933-018-0742-8

**Published:** 2018-07-04

**Authors:** Dalia El-Lebedy

**Affiliations:** Department of Clinical and Chemical Pathology, Medical Research Division, National Research Center, Al-Bohouth Street, Cairo, 12311 Egypt

**Keywords:** *CETP* Taq1B, *ANGPTL8* c194C>T, *NOS3* G894T, CVD, T2DM

## Abstract

**Background:**

The aim of the present study was to examine the association of angiopoietin-like proteins-8 (*ANGPTL8*) rs2278426, cholesteryl ester-transfer protein (*CETP*) rs708272 and endothelial nitric oxide synthase (*NOS3*) rs1799983 variants with type 2 diabetes mellitus (T2DM) and cardiovascular disease (CVD), and to investigate the effect of the potential interaction between these variants on disease risk.

**Methods:**

Our study included 272 subjects classified into 68 patients with T2DM, 68 patients with T2DM complicated with CVD and 136 control subjects. *ANGPTL8* c194C>T, *CETP* Taq1B and *NOS3* G894T polymorphisms were genotyped using TaqMan^®^ SNP Genotyping Assay.

**Results:**

The presence of *NOS3*, *ANGPTL8*, and homozygous *CETP* B1 variants were associated with increased risk of T2DM by 3.07-, 2.33- and 1.75-fold, respectively. *NOS3* variant was associated with 3.08-fold increased risk of CVD (95% CI 1.70–5.60), while *ANGPTL8* C allele was associated with 2.8-fold increased risk of CVD in T2DM patients (95% CI 1.13–6.97). Concomitant presence of both, *CETP* B1 and *NOS3* T allele, associated with increased risk of T2DM, CVD and CVD in T2DM by 8.36-, 6.33- and 7.87-fold, respectively, while concomitant presence of *ANGPTL8* variant with either *CETP* B1 or *NOS3* T allele was not associated with increased risk of T2DM or CVD. However, concomitant presence of the three variants together elevated the risk of T2DM by 13.22-fold (p = 0.004), CVD risk by 8.86-fold (p = 0.03) and highly elevated the risk of CVD in T2DM patients by 13.8-fold (p = 0.008).

**Conclusions:**

Concomitant presence of *CETP* B1, *NOS3* T and *ANGPTL8* T alleles augments the risk of CVD and T2DM. Further studies to clarify the mechanism of gene–gene interaction in the pathogenesis of CVD and T2DM are needed.

## Background

Cardiovascular disease (CVD) is the most common cause of morbidity and mortality among diabetic patients. Several factors such as dyslipidemia, obesity, smoking, exercise, alcohol intake, oxidative stress and genetic variants have been identified as risk factors for CVD in type 2 diabetes (T2DM) [1–4].

Plasma lipids and lipoproteins have been powerful risk factors for predicting CVD [4, 5]. Reduced circulating levels of high-density lipoprotein cholesterol (HDL-C) and elevated levels of very low-density lipoprotein cholesterol (VLDL-C), triglycerides (TGs) and total cholesterol (TC) are lipoprotein disorders linked to insulin resistance and atherogenesis [6].

Angiopoietin-like proteins (ANGPTLs) play major roles in lipid trafficking and metabolism. ANGPTL8 (also known as betatrophin) is a recently identified protein that is primarily expressed in liver and adipose tissue and circulates in human plasma. Expression of ANGPTL8 was found to be reduced by fasting and increased by refeeding in both mice and humans [7]. ANGPTL8 regulates the activity of lipoprotein lipase (LPL), a key enzyme in lipoprotein lipolysis pathway, through direct interaction with ANGPTL3 (the interaction partner for ANGPTL8) modulated by insulin [8]. ANGPTL8 has been associated with two functionally important processes in the development of T2DM, insulin resistance and lipid metabolism, and has been also reported to regulate the replication of β-cells in response to insulin resistance [9, 10].

Overexpression of ANGPTL8 leads to increased plasma levels of TAG (triacylglycerol) and lipoproteins via inhibition of LPL activity [11, 12]. Therefore, ANGPTL8 has been suggested as a potential therapeutic target for dyslipidemia and inhibition of ANGPTL8 has been highlighted as a novel therapeutic strategy for reducing plasma lipoprotein levels [8, 13].

Association of ANGPTL8 with diabetes and atherosclerotic diseases has been of great interest in recent studies. c194C>T (rs2278426, Arg59Trp) is a common studied variant in *ANGPTL8* gene located on chromosome 19 resulting from substitution of C for T at c194 that affects levels of the activated form of ANGPTL3 with similar effect to complete ANGPTL3 deficiency and associated with low plasma levels of LDL-C, HDL-C via relieving the inhibition of LPL [8].

Another protein, cholesteryl ester-transfer protein (CETP), plays a crucial role in the metabolism of HDL-C. CETP participates in reverse cholesterol transport by transferring cholesteryl esters from HDL-C to apolipoprotein-B containing lipoproteins such as VLDL and LDL, thereby reducing the concentration of HDL-C and alter the susceptibility to atherosclerotic vascular diseases [14–16]. *CETP* rs708272 is a single nucleotide polymorphism (SNP) in *CETP* gene located on chromosome 16q21 resulting from G to A substitution at nucleotide 277 (G277A) which disrupts TaqI restriction site. The allele containing the TaqI endonuclease site is called B1, while the allele without the restriction site is called B2 and is associated with increased HDL-C levels and decreased CETP activity and levels [17, 18]. The association of *CETP* TaqIB variant with T2DM and CAD has been investigated in several studies and gave inconsistent results among different ethnic groups [14, 19–22].

Nitric oxide (NO) is an important regulator of vasodilator tone and blood pressure, NO is formed from the oxidation of its precursor l-arginine to l-citrulline by a family of NO synthases (NOSs). NOS system consists of three distinct isoforms, encoded by three distinct genes: neuronal (*nNOS* or *NOS*-*1*), inducible (*iNOS* or *NOS*-*2*), and endothelial (*eNOS* or *NOS*-*3*) [23]. Glu298Asp (G894T, rs1799983) is one of the most clinically important polymorphisms of *NOS*-*3* gene located on chromosome 7q35-36 resulting from G to T substitution at nucleotide 894 and is associated with reduced NO production and has been suggested to play a role in the development of hypertension, atherosclerosis and CVD [24].

The aim of the present study is to examine the association of *ANGPTL8* rs2278426, *CETP* rs708272 and *NOS3* rs1799983 variants with risk of T2DM and CVD, and to assess the modulatory effect of the potential interaction between these variants on disease risk.

## Materials and methods

### Subjects

The study included 136 T2DM patients with and without CVD and 136 control subjects. All participants were recruited from the Outpatients Clinic of the National Research Center. Data of family and medical history was obtained by questionnaire. Clinical examination including measurement of systolic blood pressure (SBP) and diastolic blood pressure (DBP) was applied. Anthropometric measurements (weight and height) were collected and used for BMI calculation according to the standard formula BMI = weight (kg)/[height(m)]^2^. Hypertension was defined as blood pressure above 140/90 mmHg or taking antihypertensive drugs. Diagnosis of diabetes based on the criteria of the American Diabetes Association [25]. Studied subjects were classified into 3 groups:

*Control group* included 136 healthy subjects with fasting plasma glucose (FPG) < 100 mg/dL. Exclusion criteria were diabetes mellitus, CVD, family history of diabetes or any form of CVD, hyperlipidemia, hypertension, systemic diseases, and those under medication.

*T2DM patients without CVD* included 68 subjects fulfilled diabetes mellitus diagnostic criteria or under diabetes medication (oral and/or insulin) with no history or signs of any form of CVD.

*T2DM complicated with CVD* included 68 subjects diagnosed to have diabetes and complicated with any CVD e.g. ischemic heart disease (IHD), macroangiopathy and/or cerebrovascular disease. Exclusion criteria for diabetic patients included family history of DM or any form of CVD, and systemic diseases.

### Biochemical markers

Venous blood samples were collected from all participants after 12 h fast. Total cholesterol (TC), triglycerides (TG), high density lipoprotein cholesterol (HDL-C), low density lipoprotein cholesterol (LDL-C), fasting plasma glucose (FPG) were assayed on Roche Diagnostics clinical chemistry auto analyzer c311 (Germany). Glycosylated hemoglobin (HbA1c) was measured by high-performance liquid chromatographic (HPLC) method using Agilent 1200 HPLC system (Agilent Technologies, Germany).

### Genotyping analysis

Genomic DNA was extracted from 2 mL of whole peripheral blood using QIAamp DNA extraction kit (Qiagen Hilden, Germany, Cat No. 51304) according to the manufacturer’s protocol. *ANGPTL8* rs2278426, *CETP* rs708272 and *NOS3* rs1799983 SNPs were genotyped using TaqMan^®^ SNP Genotyping Assays. All primers and probes were designed by Applied Biosystems (Foster City, CA, USA) and genotyping analyses were performed on ABI 7500 Real Time PCR system (Applied Biosystems) according to the manufacturer’s protocol. For genotyping quality control, negative controls were included in all SNPs and 10% of samples were randomly selected and analyzed in duplicates and the concordance rate was 100%.

### Statistical analysis

Data were analyzed using IBM SPSS version 20.0 software (Statistical Package for Social Science). Quantitative data were expressed as mean values ± standard deviation (SD) and qualitative data were expressed as frequency (%). Normally distributed data were compared using Student’s t test for 2 groups and ANOVA test for more than 2 groups. The significance of differences between proportions was tested by the Chi square test (χ^2^). Differences were considered significant with p value < 0.05. Genotype and allele frequencies between groups were compared by Chi square test. Univariate logistic regression analysis was used to test the association between studied polymorphisms and diseases and were presented as unadjusted odds ratios (OR) with confidence interval (95% CI). Multivariate logistic regression analysis was used to estimate independent risk for disease development after adjustment for potential covariates age, gender, hypertension, BMI, smoking status, duration of diabetes and lipid parameters and was presented as adjusted ORs.

## Results

### General characteristics and biochemical variables of the studied participants

The study included 272 subjects classified into 68 patients with T2DM, 68 patients with T2DM + CVD, and 136 control subjects. Their age ranged from 43 to 71 years. The frequencies of CVDs in our patients were: 60% ischemic heart disease (IHD), 15% cerebrovascular disease, 15% macroangiopathy, 5% combined IHD and cerebrovascular disease, and 5% combined macroangiopathy and cerebrovascular disease.

Plasma glucose, HbA1C, BMI, SBP and DBP levels were significantly higher in patients compared to controls. The incidence of CVD among diabetic patients associated with longer durations of diabetes (p = 0.002), hypertension (p = 0.001) and smoking (p = 0.03) when compared to T2DM patients. Significantly higher levels of TC, TG and LDL-C and lower levels of HDL-C were demonstrated in patients compared to controls and in CVD patients compared to T2DM patients. Demographic, clinical and biochemical data of enrolled subjects are summarized in Table 1.

**Table 1:** Demographic, clinical and biochemical data of the studied participants

Variable	Controls (n = 136)	T2DM (n = 68)	T2DM + CVD (n = 68)
Age (years)	52.3 ± 2.5	51.8 ± 4.3	55.3 ± 5.7
Sex (male/female)	76/60	35/33	42/26
BMI (kg/m^2^)	26.1 ± 3.8	27.2 ± 2.7*	27.6 ± 2.2**
SBP (mmHg)	112 ± 8.5	125 ± 12	141 ± 20**^,†^
DBP (mmHg)	74 ± 7.5	87 ± 12	88 ± 10**^,†^
Hypertension (%)	–	30	92^†^
Smokers (%)	8	5.8	11.8^†^
Diabetes duration (years)	–	7.5 ± 4.5	12.5 ± 3.4^†^
FPG (mg/dL)	82 ± 7.5	135 ± 59*	166 ± 61**
HbA1c (%)	5.7 ± 0.5	6.8 ± 2.1*	6.9 ± 1.9**
TG (mg/dL)	127 ± 31	155 ± 70*	161 ± 56**^,†^
TC (mg/dL)	165 ± 20	178 ± 51*	200 ± 45**^,†^
LDL-C (mg/dL)	100 ± 22	120 ± 35.5*	126 ± 30**^,†^
HDL-C (mg/dL)	51.8 ± 7.9	48.5 ± 10.8*	44.1 ± 12.2**^,†^

*BMI* body mass index, *SBP* systolic blood pressure, *DBP* diastolic blood pressure, *FPG* fasting plasma glucose, *HbA1c* hemoglobin A1C, *TG* triglyceride, *TC* total cholesterol, *LDL-C* low density lipoprotein cholesterol, *HDL-C* high density lipoprotein cholesterol

* Significant p in comparison between controls and T2DM

** Significant p in comparison between controls and T2DM + CVD

^†^Significant p in comparison between T2DM and T2DM + CVD

### Association of *ANGPTL8*, *CETP*, *NOS3* polymorphisms with the risk of T2DM

For *ANGPTL8* c194C>T (rs2278426), the frequency of the variant allele (T) was significantly higher in T2DM patients than in control subjects (16% vs. 7.4%, p = 0.001) and was associated with 2.33-times increased risk of T2DM (95% CI 1.33–4.08) (Table 2). The frequency of T allele-genotypes (C/T and T/T) were significantly higher in T2DM patients than in controls under the additive model (26.5 and 3% vs. 12 and 1%, respectively, p = 0.003) and under the dominant model (29.5% vs. 13%, respectively, p = 0.002) (Table 3).

**Table 2:** Association of *NOS3*, *ANGPTL8* and *CETP* SNPs with T2DM

Gene	SNP	Cases MAF	Controls MAF	p	OR (95% CI)
*NOS3*	rs1799983	21.4	8.0	< 0.0001*	3.07 (1.82–5.19)
*ANGPTL8*	rs2278426	16	7.4	0.001*	2.33 (1.33–4.08)
*CETP*	rs708272	33	41	0.05	0.7 (0.49–1.00)

*MAF* minor allele frequency (defined based on frequency in controls)

* Significant p

**Table 3:** Association of studied polymorphisms with the risk of T2DM compared to control under different genetic models

SNP	Genotype distribution^a^		Additive model		Dominant model		Recessive model	
	Controls N = 136	Patients N = 136	OR (95% CI)	p	OR (95% CI)	p	OR (95% CI)	p
*ANGPTL8*								
rs2278426								
C/C	118 (87)	96 (70.5)	1.0 (reference)	–				
C/T	16 (12)	36 (26.5)	2.7 (1.33–3.91)	0.003*	2.73 (1.47–5.06)	0.002*	0.49 (0.08–2.73)	0.68
T/T	2 (1)	4 (3)	2.4 (0.44–13.71)		(C/C vs. C/T + T/T)		(C/C + C/T vs. T/T)	
*CETP*								
*rs708272*								
GG	44 (32)	62 (45.6)	1.0 (reference)	–				
GA	72 (53)	58 (42.6)	0.57 (0.34–0.96)	0.08	1.75 (1.07–2.86)	0.03*	1.29 (0.63–2.61)	0.29
AA	20 (15)	16 (11.8)	0.56 (0.26–1.21)		(G/G/vs. G/A + A/A)		(G/G + G/A vs. A/A)	
*NOS3*								
rs1799983								
GG	117 (86)	84 (61.8)	1.0 (reference)	–				
GT	16 (11.7)	46 (33.8)	4.0 (2.12–7.55)	< 0.001*	0.26 (0.14–0.47)	< 0.0001*	0.48 (0.11–1.99)	0.3
TT	3 (2.3)	6 (4.4)	2.78 (0.67–11.45)		(G/G vs. G/T + T/T)		(G/G +G/T vs. T/T)	

* Significant p

^a^Data shown as number of subjects (frequency)

For *CETP* G277A (rs708272), the frequency of B2 variant (allele A) was higher in controls than in T2DM patients (41% vs. 33%, p = 0.05) (Table 2). The frequency of the wild G/G genotype (B1B1) was significantly higher in T2DM patients than in control subjects compared to G/A + A/A genotypes (B1B2 + B2B2 variants, respectively) under the dominant genetic model (p = 0.03) and was associated with 1.75-fold increased risk of T2DM (95% CI 1.07–2.86). The frequency difference was of no statistical significance under the additive model (G/G vs. GA vs. A/A) (p = 0.08) or the recessive model (G/G + G/A vs. A/A) (p = 0.29) (Table 3).

For *NOS3* G894T (rs1799983), the frequency of the variant allele (T) was significantly higher in T2DM patients than in control subjects (21.4% vs. 8%, p < 0.0001) and associated with 3.07-times increased risk to develop T2DM (95% CI 1.82–5.19) (Table 2). The frequencies of T allele-genotypes (G/T and T/T) were significantly higher in T2DM patients than in controls under the additive genetic model (33.8 and 4.4% vs. 11.7 and 2.3%, respectively, p < 0.0001) and under the dominant model (38.2% vs. 14%, respectively, p < 0.0001) (Table 3).

### Association of *ANGPTL8*, *CETP*, *NOS3* polymorphisms with CVD risk

Association studies of *ANGPTL8*, *CETP* and *NOS3* variants in CVD patients compared to control subjects showed that *NOS3* T allele was significantly higher in CVD patients than in control subjects (22% vs. 8%, p = 0.0001) and was associated with 3.08-times increased risk of CVD (95% CI 1.70–5.60). Though the frequency *ANGPTL8* and *CETP* variant alleles was higher in control subjects than in CVD patients but the difference was not statistically significant (Table 4). No other significant differences were found between CVD patients and control subjects regarding other gene variants under any genetic model (Table 5).

**Table 4:** Association of *NOS3*, *ANGPTL8* and *CETP* SNPs with CVD

Gene	SNP	Cases MAF	Controls MAF	p	OR (95% CI)
*NOS3*	rs1799983	22.0	8.0	0.0001*	3.08 (1.70–5.60)
*ANGPTL8*	rs2278426	5.1	7.4	0.28	0.55 (0.21–1.42)
*CETP*	rs708272	33.8	41	0.9	1.0 (0.64–1.54)

*MAF* minor allele frequency (defined based on frequency in controls)

* Significant p

**Table 5:** Association of studied polymorphisms with the risk of CVD compared to control under different genetic models

SNP	Genotype distribution^a^		Additive model		Dominant model		Recessive model	
	Controls N = 136	Patients N = 68	OR (95% CI)	p	OR (95% CI)	p	OR (95% CI)	p
*ANGPTL8* rs2278426								
C/C	118 (87)	62 (91)	1.0	–				
C/T	16 (12)	5 (8)	0.70 (0.26–1.91)	0.79	1.35 (0.53–3.40)	0.3	1.01 (0.09–11.39)	0.7
T/T	2 (1)	1 (1)	0.95 (0.08–10.70)		(C/C vs. C/T + T/T)		(C/C + C/T vs. T/T)	
*CETP* rs708272								
GG	44 (32)	32 (47.1)	1.0	–				
GA	72 (53)	26 (38.2)	0.6 (0.32–1.14)	0.07	1.34 (0.75–2.42)	0.2	0.4 (0.18–1.16)	0.08
AA	20 (15)	10 (14.7)	1.68 (0.63–4.49)		(G/G/vs. G/A + A/A)		(G/G + G/A vs. A/A)	
*NOS3* rs1799983								
GG	117 (86)	41 (60.3)	1.0	–				
GT	16 (11.7)	24 (35.3)	4.09 (1.98–8.47)	0.0001*	0.25 (0.12–0.49)	< 0.0001*	2.05 (0.40–10.41)	0.6
TT	3 (2.3)	3 (4.4)	2.73 (0.53–14.08)		(G/G vs. G/T + T/T)		(G/G +G/T vs. T/T)	

* Significant p

^a^Data shown as number of subjects (frequency)

### Association of *ANGPTL8*, *CETP*, *NOS3* polymorphisms with CVD risk in T2DM

The frequency of *ANGPTL8* C allele (wild allele) was significantly higher in T2DM + CVD patients than in T2DM patients (96.3% vs. 86.7% p = 0.03) and associated with 2.8-times increased risk of CVD in T2DM patients (95% CI 1.13–6.97), while the variant T allele was significantly higher in T2DM patients without CVD than in T2DM + CVD patients (13.3% vs. 3.7%, respectively). There was no other statistical significant difference regarding alleles frequency and genotypes distribution of *CETP* and *NOS3* variants between T2DM patients and T2DM + CVD patients under any genetic model (Table 6).

**Table 6:** Associations of studied polymorphisms with the risk of CVD in T2DM

SNP	Genotype distribution^a^		Additive model		Dominant model		Recessive model	
	T2DM (n = 68)	T2DM + CVD (n = 68)	OR (95% CI)	p	OR (95% CI)	p	OR (95% CI)	p
*ANGPTL8* rs2278426								
C/C	53 (78)	62 (91.2)	1.0 (reference)	–				
C/T	12 (17.6)	5 (7.4)	2.8 (0.92–8.48)	0.15	0.34 (0.12–0.94)	0.05	0.32 (0.03–3.1)	0.6
T/T	3 (4.4)	1 (1.4)	3.50 (0.35–34.74)		(C/C vs. C/T + T/T)		(C/C + C/T vs. T/T)	
^†^Allele C	118 (86.7)	129 (96.3)	2.8 (1.13–6.97)	0.03*				
Allele T	18 (13.3)	7 (3.7)						
*CETP* rs708272								
GG	30 (44)	32 (47.1)	1.0	–				
GA	32 (47)	26 (38.2)	0.7(0.37–1.56)	0.4	0.88 (0.45–1.74)	0.8	1.78 (0.60–5.21)	0.4
AA	6 (9)	10 (14.7)	0.6 (0.20–1.97)		(G/G/vs. G/A + A/A)		(G/G + G/A vs. A/A)	
Allele G	92 (67.6)	90 (66.2)	1.06 (0.64–1.77)	0.89				
Allele A	44 (32.4)	46 (33.8)						
*NOS3* rs1799983								
GG	44 (67.4)	41 (60.3)	1.0 (reference)	–				
GT	20 (29.4)	24 (35.3)	0.7 (0.37–1.61)	0.7	0.82 (0.41–1.66)	0.7	1.35 (0.29–6.29)	1.0
TT	4 (5.9)	3 (4.4)	1.37 (0.29–6.39)		(G/G vs. G/T + T/T)		(G/G +G/T vs. T/T)	
Allele G	108 (79.4)	106 (78)	0.91 (0.51–1.63)	0.88				
Allele T	28 (20.6)	30 (22)						

* significant p

^†^Alleles frequency calculated in 2N

^a^Data shown as number of subjects (%)

### Synergistic effect of *ANGPTL8*, *CETP and NOS3* variants on the risk of T2DM and CVD

Studying the synergistic effect of *CETP* B1, *NOS3* T and *ANGPTL8* T alleles on the risk of CVD and T2DM showed that the concomitant presence of *CETP* B1 and *NOS3* T allele, was associated with 8.36-fold increased risk of T2DM (p < 0.0001), 6.33-fold increased risk of CVD (p = 0.0002) and 7.87-fold increased risk of CVD in T2DM patients (p = 0.006). Concomitant presence of *ANGPTL8* T allele with either *CETP* B1 or *NOS3* T allele was not associated with increased risk of T2DM or CVD. Meanwhile, concomitant presence of *CETP* B1, *NOS3* T and *ANGPTL8* T alleles together elevated the risk of T2DM by 13.22-fold (p = 0.004) and CVD risk by 8.86-fold (p = 0.03) and highly elevated the risk of CVD in T2DM patients by 13.8-fold (p = 0.008) (Table 7).

**Table 7:** Synergistic effect of *NOS3* T, *CETP* B1 and *ANGPTL8* T alleles on the risk of T2DM and CVD

*NOS3* T	*CETP* B1	*ANGPTL8* T	Total T2DM patients % OR (95% CI) p-value	T2DM without CVD^a^ % OR (95% CI) p-value	T2DM + CVD % OR (95% CI) p-value	Control %
−	−	−	41.7	44.5	50	64.3
−	+	+	8.3	5.5	5.5	28.5
			0.4 (0.17–1.10) p = 0.07	0.69 (0.08–5.46) p = 0.7	0.19 (0.04–0.88) p = 0.3	
+	+	−	39	39	33.3	7.2
			8.36 (3.35–20.87) p < 0.0001**	7.87 (1.54–40.09) p = 0.006**	6.33 (2.29–17.46) p = 0.0002**	
+	−	+	2.7	0	5.7	0
			1.88 (0.11–31.09) p = 0.9	4.25 (0.25–71.6) p = 0.35	5.9 (0.51–68.29) p = 0.17	
+	+	+	8.3	11	5.5	0
			13.22 (1.56–111.7) p = 0.004**	13.8 (1.63–116.5) p = 0.008**	8.86 (0.87–89.54) p = 0.03**	

Data shown as frequency (%)

**Significant p

^a^T2DM patients vs. T2DM + CVD

## Discussion

Individuals with T2DM are at an increased risk of CVD and glycemic control is not enough to halt the progression of vascular complications [26–28]. Both genetic and environmental factors have important roles in the pathogenesis of T2DM and CVD [29] and the role of genetic factors appears to be considerably different between various populations [30].

In this work, we studied the association of *CETP* rs708272, *NOS3* rs1799983 and *ANGPTL8* rs2278426 variants with risk of CVD and T2DM in our population. Our results showed that *NOS3* rs1799983, *ANGPTL8* rs2278426 and *CETP* rs708272 variants associated with increased risk of T2DM by 3.07-, 2.33- and 1.75-fold, respectively, while only *NOS3* variant associated with 3.08-fold increased risk of CVD. Meanwhile, ANGPTL8 wild allele (C) associated with 2.8-fold elevated risk of CVD in T2DM patients, while the variant allele (T) had a protective role against the development of CVD.

*NOS3* gene has a role in maintaining normal endothelial function by coding for endothelial NOS enzyme which through synthesis of NO affects the relaxation of vascular smooth muscle, inhibits adhesion of platelets and leukocytes to the endothelium, reduces vascular smooth muscle cell migration and proliferation, and limits the oxidation of LDL-C [31, 32]. Reduced production of NO is one of the most important contributors to endothelial dysfunction and an important initiator of vascular complications [33].

In a recent study to identify the cellular mechanisms by which advanced glycation end-products (AGEs) exacerbate the endothelial dysfunction in human coronary artery endothelial cells (HCAECs) in diabetic patients with coronary artery atherosclerosis, AGEs were associated with increased oxidative stress and with significant reduction of eNOS mRNA and protein levels, eNOS mRNA stability, eNOS enzyme activity, and cellular NO levels [34].

G894T polymorphism in the *NOS3* gene, which is associated with reduced NOS enzyme activity and consequently bioavailability of NO, has been associated with diabetes and diabetes related traits [35, 36], as well as relevant sub-clinical cardiac remodeling in CKD (chronic kidney disease) and has been reported as an important genetic biomarker in non-dialysis CKD patients who are at risk of worsening cardiac disease with progression of renal dysfunction [37].

Our findings are consistent with several reports that have indicated an association between *NOS3* G894T and T2DM and CVD [35, 36, 38, 39]. However, some studies lack such association [40–42]. In addition to its endothelial functions, NOS3 has been indicated as an independent determinant of DNA methylation. In a previous study measuring global DNA methylation in individuals with different levels of glucose tolerance in parallel with genetic screening of polymorphisms in *NOS3* gene, global DNA methylation was increased in both pre-diabetic and diabetic individuals after adjustment for other factors such as age, gender, smoking, and glucose tolerance status, and was associated with *NOS3* G894T polymorphism [43]. In a study involving obese children and type 1 diabetes with *NOS3* polymorphism demonstrated an improved endothelial function after supplementation with folate [44] which is known to play an important role in DNA methylation and reduced folate level has been linked to aberrant DNA methylation profiles and epigenetic alterations [45, 46]. However, it is still unclear how NOS3 may influence global DNA methylation.

On the other hand, ANGPTL8 has been linked to insulin resistance and lipoprotein metabolism, two functionally important processes in the development of type 2 diabetes and CVD [47]. In recent studies, association of ANGPTL8 with diabetes and atherosclerotic diseases has gained much interest. ANGPTL8 leads to increased plasma level of lipoproteins via inhibition of LPL activity, rs2278426 variant affects the level of the activated form of ANGPTL3, the interaction partner for ANGPTL8, therefore affects the function of ANGPTL8 and associated with low plasma levels of LDL-C and HDL and reduced risk of CVD in humans [8, 48].

Circulating level of ANGPTL8 was significantly higher in subject with MetS (metabolic syndrome) as well as subjects with increasing number of MetS components such as insulin resistance and central obesity. ANGPTL8 also showed significant association with hsCRP, BMI, TG, LDL, HOMA-IR and FPG and was associated with 2.4-fold increase of having MetS [49]. While some studies reported increased levels of ANGPTL8 in obeity and T2DM [50–52] and being associated with C-peptide production in non-diabetic subjects [53], other studies showed that ANGPTL8 was reduced in T2DM [54, 55].

Ethnic variation has been associated with variable minor allele frequency (MAF) rates for this variant in respect to LDL-C and HDL-C plasma levels. In Dallas Heart Study (DHS), a multiethnic population-based study of Dallas County, Hispanics had the highest MAF of 26%, followed by African-Americans (18%) and the least was reported in European-Americans (5%). A*NGPTL8* rs2278426 was associated with lower LDL-C and HDL-C in African-Americans and Hispanics but not in European-Americans, and was not associated with fasting glucose or homeostatic model assessment-insulin resistance in any ethnic group [56]. However, in a genome-wide association study that predominantly included individuals of European ancestry, this variant was associated with both HDL-C and LDL-C [57]. Meanwhile, in a study on non-diabetic Arabs, individuals who carried this variant had higher FPG, but did not show significant difference in their LDL and HDL levels compared to the wild type carriers [58]. Similar findings were observed by Quagliarini et al. [8] in Hispanic participants of the DHS but not in African Americans or Europeans as Hispanic subjects who were homozygous for the variant allele had significantly higher FPG than subjects who were homozygous for the wild allele, however, this was only observed in a very small number of participants which was ignored in their discussion.

The role of ANGPTL8 in beta-cell proliferation has been questioned and the exact mechanism for the effect of this variant has not been elucidated yet. It’s possible to speculate that the change in the amino acid sequence might affect ANGPTL8 protein structure which might disrupt its functional domains [59, 60], however the functional impact of this polymorphism on the function of ANGPTL8 requires further investigation. Our data highlights the importance of ethnicity in understanding the role of ANGPTL8 and suggest a role for ANGPTL8 in glucose metabolism in our population.

Cholesteryl ester-transfer protein is another key regulating factor of lipid metabolism, therefore, polymorphisms of its gene may be candidates for modulating the lipid parameters and altering the susceptibility to atherosclerotic diseases [22]. TaqIB is the most studied polymorphism of *CETP* gene with a frequency of 0.44 in Caucasian populations, this variant associates with decrease in CETP activity and level, increase in HDL-C level and modulated risk for diabetic complications. The presence of homozygous B1 allele associates with the lowest HDL-C levels while homozygous B2 allele associates with the highest levels [18, 61].

The role of TaqIB polymorphism in metabolic syndrome, T2DM and CAD, either through its influence on lipid metabolism or independent to its effect on lipid profile, has been reported in various populations [14, 19–22, 62]. In the present study, the presence of homozygous B1 allele increased the risk of T2DM in our population by 1.75-fold, but we could not report a significant association with the CVD. In a meta-analysis performed on 13,677 subjects, TaqIB variant exhibited a highly significant association with HDL-C levels and CAD, B2B2 individuals had significantly higher HDL-C levels than B1B1 individuals [14]. In Tunisian population, B1 allele was associated with lower concentration of HDL-C, decreased CETP activity and increased prevalence of CAD in T2DM patients [19]. In Spanish population the GG (B1B1) genotype carriers showed significantly lower HDL-C concentrations than the B2-allele (A) carriers, as well as higher glucose levels after the oral glucose tolerance test [63]. Also, B1 allele of CETP has been associated with the risk of CAD and T2DM independent to HDL-C level [64] and was reported as an independent risk factor and a strong genetic predictor of macrovascular complications in T2DM [22]. Inhibitors of CETP is under current investigation as potential drugs for reducing cardiovascular disease. Animal studies, as well as clinical and epidemiologic evidence, have suggested that inhibiting CETP is an effective strategy for raising HDL-C levels and reducing LDL-C levels [65].

In the present work, we examined the influence of concomitant presence of *CETP* B1, *NOS3* T and *ANGPTL8* T alleles on the risk of CVD and T2DM. We demonstrated elevated risk of T2DM, CVD and CVD in T2DM in the presence of both *CETP* B1 and *NOS3* T alleles to the level of 8.36-, 6.33-, and 7.87-fold, respectively. Concomitant presence of the three variants together elevated the risk of T2DM and CVD by 13.22- and 8.86-fold, respectively and highly elevated the risk of CVD in T2DM patients by 13.8-fold. Our results indicate that the presence of variations in more than one gene play an important role in the susceptibility to T2DM and CVD. The limitations of our study that it was a cross sectional study and the small sample size. The exact mechanism of the influence of interaction between these variants on the risk of CVD and T2DM needs further investigations.

## Conclusion

Concomitant presence of *CETP* B1, *NOS3* T and *ANGPTL8* T alleles augments the risk of CVD and T2DM in our population. The results of the present study emphasize the role of gene–gene interaction in the pathogenesis of complex disorders such as CVD and T2DM and the underlying mechanisms need to be clarified in future studies.

## Abbreviations

CVD: cardiovascular disease
T2DM: type 2 diabetes mellitus
ANGPTL: angiopoietin-like protein
ANGPTL8: angiopoietin-like proteins-8
ANGPTL3: angiopoietin-like proteins-3
CAD: coronary artery disease
BMI: body-mass index
SBP: systolic blood pressure
DBP: diastolic blood pressure
FPG: fasting plasma glucose
TC: total cholesterol
TG: triglycerides
LDL: low-density lipoprotein
HDL: high-density lipoprotein
VLDL: very low density lipoprotein
HbA1c: glycosylated hemoglobin
IHD: ischemic heart disease
HPLC: high-performance liquid chromatographic
PCR: polymerase chain reaction
SNP: single nucleotide polymorphism
LPL: lipoprotein lipase
TAG: triacylglycerol
CETP: cholesteryl ester transfer protein
NO: nitric oxide
NOS: NO synthase
nNOS: neuronal NOS
iNOS: inducible
eNOS: endothelial NOS
DHS: Dallas Heart Study
AGEs: advanced glycation end-products
CKD: chronic kidney disease
MetS: metabolic syndrome
